# A Simplified Convex Optimization Model for Image Restoration with Multiplicative Noise

**DOI:** 10.3390/jimaging9100229

**Published:** 2023-10-20

**Authors:** Haoxiang Che, Yuchao Tang

**Affiliations:** 1 Department of Mathematics, Nanchang University, Nanchang 330031, China; 405500210011@email.ncu.edu.cn; 2 School of Mathematics and Information Science, Guangzhou University, Guangzhou 510006, China

**Keywords:** multiplicative noise removal, total variation regularization, ADMM, convex variational model, 68U10, 65K10, 65K15, 35A15, 94A08

## Abstract

In this paper, we propose a novel convex variational model for image restoration with multiplicative noise. To preserve the edges in the restored image, our model incorporates a total variation regularizer. Additionally, we impose an equality constraint on the data fidelity term, which simplifies the model selection process and promotes sparsity in the solution. We adopt the alternating direction method of multipliers (ADMM) method to solve the model efficiently. To validate the effectiveness of our model, we conduct numerical experiments on both real and synthetic noise images, and compare its performance with existing methods. The experimental results demonstrate the superiority of our model in terms of PSNR and visual quality.

## 1. Introduction

Image restoration is a fundamental problem in image processing that focuses on recovering the original image from a degraded version. It encompasses various problems like image denoising, image deblurring, and image inpainting, and many others. For image denoising, there are different types of noise that can corrupt the image, including additive Gaussian noise, Poisson noise, and impulse noise. These noise types are commonly encountered and well-studied in the field of image processing. Several restoration algorithms have been developed to address these types of noise and achieve better image quality. However, multiplicative noise is distinct from these commonly encountered noise types. It deviates from the traditional assumption of additive noise and follows a different statistical distribution, such as the Gamma or Rayleigh distribution. Multiplicative noise can arise in various imaging scenarios, such as synthetic aperture radar (SAR) [1], ultrasound [2], laser images [3], and other coherent image systems [4]. The mathematical model for image degradation caused by blur and multiplicative noise can be expressed as:

(1) f=(Au)∘η,

where $f\in R^{m}$ represents the observed blurred and noisy image, $A\in R^{m\times m}$ is the blurring operator, and $u\in R^{m}$ represents the original image that to be recovered. The symbol ∘ denotes the element-wise product between two vectors, and η represents the multiplicative noise interference. When the blurring operator *A* is an identity matrix (A=I), the problem degenerates into a multiplicative noise denoising problem. Without the loss of generality, it is assumed that both *u* and η are greater than 0, implying that *f* is also greater than 0. The objective of this inverse problem is to reconstruct the original image that has been distorted by multiplicative noise η. However, solving this problem is challenging due to its ill-posed nature, wherein a unique or stable solution does not exist. Consequently, regularization techniques are necessary to obtain a reasonable approximation of the original image.

The problem of multiplicative noise has received considerable attention in past research. Various methods have been proposed in the literature to address this issue, such as the filter-based method [5], wavelet-based method [6,7], hybrid methods [8], and variational-based methods. Variational-based methods are a class of image restoration techniques that use variational calculus to formulate and solve the inverse problem of recovering a clean image from a noisy one. They often involve minimizing an energy functional that consists of a data fidelity term and a regularization term. The data fidelity term measures how well the restored image matches the observed image, whereas the regularization term imposes some prior knowledge or smoothness constraints on the restored image. To address the problem of removing multiplicative noise, Rudin, Lions, and Osher [9] introduced a variational model that utilizes the mean and variance information of the noise. This model, commonly referred to as the RLO model, innovatively incorporates the TV regularity term to preserve the sharpness of the recovered image edges. The RLO model is defined as follows:

(2) $$\begin{array}{cc} \; & \min_{u}{∥u∥}_{TV}\\ \; & s.t.\;\frac{f}{Au}=1\\ \; & {\left(\frac{f}{Au}-1\right)}^{2}=\theta^{2}, \end{array}$$

where $\theta^{2}$ represents the variance of the noise. The RLO model only considers the mean and variance of the noise, but does not take into account its distribution. Based on the property that multiplicative noise follows a Gamma distribution with a mean of 1, Aubert and Aujol [10] proposed another variational model using the maximum a posteriori (MAP) estimation method to estimate the original image from the noisy one, which is widely known as the AA model:

(3) $$\min_{u}\langle \log\left(Au\right)+\frac{f}{Au},1\rangle +\lambda {∥u∥}_{TV},$$

where the first term in the AA model is the data fidelity term, the second term is the TV regularization term, and the regularization parameter λ balances the trade-off between the two terms in the objective function. The AA model is a non-convex optimization problem that poses challenges for finding its global solution. Several algorithms have been adapted from the literature to deal with this problem. One of them is the difference of convex algorithm (DCA) proposed by Zeng et al. [11], which transforms the energy function of the AA model into a form of the difference of two convex functions, and then alternately solves the two convex subproblems. Another approach is to use the split Bregman method, which was employed by Bioucas-Dias and Figueiredo [12,13] to handle the AA model. Woo and Hyenkyun [14] also developed a proximal linearized alternating direction (PLAD) algorithm for the same purpose. Some other methods focus on selecting spatially adaptive regularization parameters [15] or converting the multiplicative noise into additive noise [16,17].

To deal with the non-convexity of the AA model, several algorithms have been proposed in the literature. For example, Zeng et al. [11] used a difference of convex algorithm (DCA) to transform the energy function of the non-convex AA model into a convex minus convex one, and then solved for the two convex ones to obtain the solution of the AA model. Bioucas-Dias and Figueiredo utilized the split-Bregman or variable splitting method to solve the AA model [12,13]. Woo and Hyenkyun [14] also developed the proximal linearized alternating direction (PLAD) algorithm for the same purpose, and some papers focus on selecting spatially adaptive regularization parameters [15]. Moreover, some methods convert the multiplicative noise into additive noise, such as the Box-Cox transformation [16] and the ADMM algorithm [17].

The RLO model and the AA model are both non-convex models. Solving a non-convex model is challenging because the existence and uniqueness of the solutions are not guaranteed. The authors of the paper on the AA model prove the existence of their solution, but only give a sufficient condition for the uniqueness. The condition is that *u* satisfies 0<u<2f. This implies that the AA model is strictly convex in this case and, thus, has a unique solution. However, a general proof of uniqueness for the AA model is still lacking. Several methods have been developed to cope with the non-convexity of multiplicative noise models, especially for the AA model.

By applying a logarithmic transformation to the AA model using z=log(u), one obtains the logarithmic model. This model has been investigated by various authors, such as [18,19,20,21]. Shi and Osher [18] developed a method to handle the non-convexity in multiplicative noise by using a log transform, and obtained a general form that encompasses different models for different parameters. This model is called the SO model in the literature. Huang et al. [19] also introduced a logarithmic model known as the HNW model. The HNW model differs from the SO model in that it directly applies the logarithmic transform to the AA model. Since the logarithmic model is convex, a unique solution exists, and after obtaining it, an exponential transformation is applied to obtain the desired recovery image. Apart from the logarithmic transformation of *u* that ensures convexity, taking the *m*th root transformation of *u* can also convert the AA model into a convex one. This approach is discussed, for example, in [22,23,24].

The logit model and the *m*th root model are only applicable to denoising tasks and cannot be used for deblurring. However, Dong and Zeng [25] addressed the non-convexity of the AA model by introducing a quadratic penalty term, enabling it to handle deblurring tasks. This model is more versatile compared to the logarithmic and mth root models, which are limited to denoising. In another approach, Steidl and Teuber [26] utilized the classical *I*-divergence to address multiplicative noise and proposed the *I*-divergence model. Additionally, Woo and Yun [14] proposed a proximal linearized alternating direction method for solving the logarithmic and I-divergence models. In contrast, Zhao et al. [27] proposed a new model based on the idea of decoupling. The main concept behind this model is to separate the image from the noise η on the right-hand side of (1) by incorporating it into the left-hand side of the equation. We refer to this model as the ZWN model:

(4) $$\min_{w,u}\frac{1}{2}{∥w-\mu e∥}_{2}^{2}+\alpha_{1}{∥Fw-Au∥}_{1}+\alpha_{2}{∥u∥}_{TV},$$

where μ is the mean value of *w*, *e* is a vector of all 1s, and $\alpha_{1}$ and $\alpha_{2}$ are two positive regularization parameters. Since the ZWN model does not use information from the noise distribution, it can be applied to multiplicative noise problems with noise distributions other than the Gamma distribution. Furthermore, Wang et al. [28] made enhancements to the ZWN model and introduced an *l*-curve method for the selection of regularization parameters.

The ZWN model has two regularization parameters, which control the trade-off between data fidelity and model complexity. However, selecting appropriate regularization parameters can be challenging. To address this issue, we propose to impose the second term $l_{1}$ norm in the ZWN model as an equality constraint on the model, i.e., Fw=Au. This approach improves the sparsity of the vector Fw-Au, so that Fw and Au are close. Sparsity is desirable because it can reduce noise and preserve edges in the restored images. Moreover, this approach reduces the number of regularisation parameters from two to one, thus simplifying the model selection process. The numerical experimental results on both real and synthetic noise images indicate that our model generally outperforms the ZWN model in terms of image quality.

The rest of this paper is organized as follows. In Section 2, we review the derivation of the ZWN model and its advantages and limitations. In Section 3, we present a new convex model based on reducing the number of regularization parameters in the ZWN model and propose numerical methods for solving it. In Section 4, we show the results of the numerical experiments on both real and synthetic noise images that are affected by multiplicative noise, and compare our model with the ZWN model and other existing methods. Finally, some conclusions are given.

## 2. Review of the ZWN Model

In this section, we briefly review the ZWN model, which was proposed by Zhao et al. [27]. The main idea of the ZWN model is to decouple *u* and η in (1) by rewriting it as:

(5) Fw=Au,

where *F* is a diagonal matrix with elements ${\left[f\right]}_{i}$, and *w* is a vector satisfying ${\left[w\right]}_{i}=1/{\left[\eta \right]}_{i}$. In this way, *u* and *w* are decoupled, and so are *u* and η. Therefore, it is naturally raise the following optimization problem with the L1 norm as the data fidelity term and the TV norm as the regularization:

(6) $$\min_{w,u}\;{∥Fw-Au∥}_{1}+\alpha {∥u∥}_{TV},\;$$

where α is a positive parameter that balances the two terms. However, this model has a trivial solution, which is u=ψ1 and $w=\psi F^{-1}A1$, where ψ is any scalar. The minimum value of the objective function in (6) is greater than zero. To avoid this situation, Zhao et al. added a quadratic term $\frac{1}{2}\left||w-\mu e\right|{|}_{2}^{2}$ to the model, resulting in (4).

Next, we review the concept of total variation (TV), which is a popular regularization term that exploits the sparsity of the gradient of images. Anisotropic total variation and isotropic total variation are two variants of the TV regularization. The difference between them is that anisotropic total variation treats horizontal and vertical gradients separately, whereas isotropic total variation combines them into a term that does not change with rotation. In detail, the anisotropic total variation is defined by:

$${∥u∥}_{TV}={∥Du∥}_{1}=\sum_{i,j}|D_{h}u_{i,j}\left|+\right|D_{v}u_{i,j}|,$$

where $D_{h}$ and $D_{v}$ are finite difference operators in horizontal and vertical directions, respectively. The isotropic total variation is defined by:

$${∥u∥}_{TV}={∥Du∥}_{2}=\sum_{i,j}\sqrt{{\left(D_{h}u_{i,j}\right)}^{2}+{\left(D_{v}u_{i,j}\right)}^{2}}.$$

TV regularization can preserve edges and remove noise in an image, but it may also introduce some artifacts, such as staircasing and the loss of fine details. To overcome these drawbacks, some variants of TV regularization have been proposed, such as weighted TV [29], nonlocal TV [30], and higher-order TV [31], etc.

## 3. The Proposed Model and Main Algorithm

This section outlines the main methodology employed in this study. The ZWN model is a convex variational model designed to restore images corrupted by multiplicative noise. It consists of three main components: the variance term, which ensures the strict convexity of the model under a mild condition; the data fidelity term, which matches the multiplicative noise model; and the regularization term, which imposes a total variation regularization on the image. $\alpha_{1}$ and $\alpha_{2}$ are two positive parameters that are used to balance the three terms. However, selecting suitable values for these parameters can be challenging and time-consuming. To address this issue and minimize the number of regularization parameters, we propose a new model that enhances the sparsity of the matrix Fw-Au by imposing an equality constraint: Fw=Au. This approach not only improves the image quality and edge preservation, but also simplifies the process of selecting model parameters. The formulation of the proposed model is as follows:

(7) $$\begin{array}{cc} \; & \min_{w,u}\frac{1}{2}{∥w-\mu e∥}_{2}^{2}+\alpha {∥u∥}_{TV}\\ \; & \;s.t.\;Fw=Au, \end{array}$$

where α is a positive regularization parameter to control the balance between the two terms in the objective function, and μ is the mean value of *w*. We can see that if A=I, then our model reduces to a convex model for multiplicative denoising. Our model is based on modifying the second term of the ZWN model into an equality constraint, as this term is l1-norm. This way our model will only have one regularization parameter, which is one less parameter compared to the ZWN model.

### Main Algorithm

To solve the proposed model (7), we can use the well known ADMM method. By introducing a auxiliary variable *p*, (7) can be written as:

(8) $$\begin{array}{cc} \; & \;\min_{w,u,p}\;\frac{1}{2}{∥w-\mu e∥}_{2}^{2}+\alpha {∥p∥}_{\beta }\\ \; & s.t.\;Fw=Au,Du=p, \end{array}$$

where β=1 or 2.

The augmented Lagrangian function associated with the problem (8) is defined as follows:

(9) $$\begin{array}{cc} \; & L_{\rho_{1},\rho_{2}}\left(w,u,p;\lambda_{1},\lambda_{2}\right)=\frac{1}{2}{∥w-\mu e∥}_{2}^{2}+\alpha {∥p∥}_{\beta }+\langle \lambda_{1},Fw-Au\rangle \\ \; & +\langle \lambda_{2},Du-p\rangle +\frac{\rho_{1}}{2}{∥Fw-Au∥}_{2}^{2}+\frac{\rho_{2}}{2}{∥Du-p∥}_{2}^{2}, \end{array}$$

where $\lambda_{1}$ and $\lambda_{2}$ are the Lagrangian multipliers and $\rho_{1}>0,\rho_{2}>0$ are the penalty parameters. Therefore, the ADMM method leads to the following subproblems:

(10) $$\left\{\begin{array}{c} w^{k+1}=\underset{w}{\arg\min}\;\frac{1}{2}{∥w-\mu e∥}_{2}^{2}+\langle \lambda_{1}^{k},Fw-Au^{k}\rangle +\frac{\rho_{1}}{2}{∥Fw-Au^{k}∥}_{2}^{2}\\ u^{k+1}=\underset{u}{\arg\min}\;\langle \lambda_{1}^{k},Fw^{k+1}-Au\rangle +\langle \lambda_{2}^{k},Du-p^{k}\rangle +\frac{\rho_{1}}{2}∥Fw^{k+1}-Au∥+\frac{\rho_{2}}{2}{∥Du-p^{k}∥}_{2}^{2}\\ p^{k+1}=\underset{p}{\arg\min}\;\alpha {∥p∥}_{\beta }+\langle \lambda_{2}^{k},Du^{k+1}-p\rangle +\frac{\rho_{2}}{2}{∥Du^{k+1}-p∥}_{2}^{2}\\ \lambda_{1}^{k+1}=\lambda_{1}^{k}+\rho_{1}\left(Fw^{k+1}-Au^{k+1}\right)\\ \lambda_{2}^{k+1}=\lambda_{2}^{k}+\rho_{2}\left(Du^{k+1}-p^{k+1}\right) \end{array}\right.$$

In the following, we will illustrate how to solve the subproblems in the ADMM scheme (10).

For the *w*-subproblem, since the equation with respect to *w* is differentiable, we can directly compute the derivative of the equation at 0:

(11) $$0=\left(w^{k+1}-\mu e\right)+F^{T}\lambda_{1}^{k}+\rho_{1}F^{T}\left(Fw^{k+1}-Au^{k}\right).$$

We can then simplify the resulting equation to obtain the solution for the *w*-subproblem:

(12) $${\left[w\right]}_{i}^{k+1}=\frac{{\left[\mu e-F^{T}\lambda_{1}^{k}+\rho_{1}F^{T}Au^{k}\right]}_{i}}{{\left[I+\rho_{1}F^{T}F\right]}_{i,i}},i=1,\cdots ,m,$$

where $F^{T}$ denotes the transpose of *F*, and the superscript *k* for each variable refers to the number of iterations of the $k^{th}$ iteration in the ADMM algorithm. The subscript *i* of a variable refers to the $i^{th}$ element in the vector, whereas the subscript i,i refers to the element in row i and column *i* of the matrix. Since we assume that the image we are considering has m∗n pixels, *i* is bounded by *m*. Here, *I* is a matrix of size m∗m with all elements being 1.

For the *u*-subproblem, we apply the same method as for the *w*-subproblem to derive an equation and simplify it, resulting in the following equation:

(13) $$\left(\rho_{1}A^{T}A+\rho_{2}D^{T}D\right)u^{k+1}=\lambda_{1}^{k}A^{T}-D^{T}\lambda_{2}^{k}+\rho_{1}A^{T}Fw^{k+1}+\rho_{2}D^{T}p^{k}.$$

Under the periodic boundary conditions for the image u, both the matrices $A^{T}A$ and $D^{T}D$ are block circulant, with circulant blocks that can be diagonalized using a 2D fast Fourier transform (FFT). Therefore, Equation (13) can be efficiently solved using one FFT and one inverse FFT, as follows:

(14) $$u^{k+1}=F^{-1}\left(\frac{F\left(A^{T}\lambda_{1}^{k}-D^{T}\lambda_{2}^{k}+\rho_{1}A^{T}Fw^{k+1}+\rho_{2}D^{T}p^{k}\right)}{\rho_{1}{\left|F\left(A\right)\right|}^{2}+\rho_{2}{\left|F\left(D\right)\right|}^{2}}\right),$$

where F(.) and $F^{-1}\left(.\right)$ represent the FFT and inverse FFT, respectively.

For the *p*-subproblem, we will divide it into two cases:

When β=2, we have:

(15) $${\left[p\right]}_{i}^{k+1}=\max\left\{{∥{\left[Du^{k+1}+\frac{\lambda_{2}^{k}}{\rho_{2}}\right]}_{i}∥}_{2}-\frac{\alpha }{\rho_{2}},0\right\}\frac{{\left(Du^{k+1}+\frac{\lambda_{2}^{k}}{\rho_{2}}\right)}_{i}}{{∥{\left[Du^{k+1}+\frac{\lambda_{2}^{k}}{\rho_{2}}\right]}_{i}∥}_{2}}.$$

When β=1, we have:

(16) $${\left[p\right]}_{i}^{k+1}=\max\left\{\left|{\left[Du^{k+1}+\frac{\lambda_{2}^{k}}{\rho_{2}}\right]}_{i}\right|-\frac{\alpha }{\rho_{2}},0\right\}sign\left({\left(Du^{k+1}+\frac{\lambda_{2}^{k}}{\rho_{2}}\right)}_{i}\right).$$

Finally, we present Algorithm 1, which outlines the ADMM method for solving the proposed model (7).
**Algorithm 1:** ADMM method for solving the proposed model (7) **Input:**$\rho_{1}>0$, $\rho_{2}>0$, α>0 **Initialize:**$u^{0}=f$, $\lambda_{1}^{0},\lambda_{2}^{0},p^{0}$  1:**while** $\frac{∥u^{k}-u^{k+1}{∥}_{2}}{∥u^{k}{∥}_{2}}>\varepsilon$ **do**  2:    Calculate $w^{k+1}$ by (12),  3:    Calculate $u^{k+1}$ by (14),  4:    Calculate $p^{k+1}$ by (15) or (16),  5:    $\lambda_{1}^{k+1}=\lambda_{1}^{k}+\rho_{1}\left(Fw^{k+1}-Au^{k+1}\right)$,  6:    $\lambda_{2}^{k+1}=\lambda_{2}^{k}+\rho_{2}\left(Du^{k+1}-p^{k+1}\right)$,  7:    k=k+1,  8:**end while**

## 4. Numerical Experiments

In this section, we present numerical results to evaluate the performance of our proposed method. We compare our approach with the AA model [10] by Aubert and Aujol; the RLO model [9] by Rudin, Lions, and Osher; and the ZWN model [27] by Zhao, Wang, and Ng. The AA model and the RLO model are solved using the projected gradient method, whereas the ZWN model is solved using the ADMM. All tests were conducted on Windows 11 and MATLAB version 9.13 (R2022b) on a laptop running AMD Ryzen 9 6900HX at 3.30 GHz with 16 GB of RAM. For simulating the Gamma distribution, we utilize the ’gamrnd’ function in MATLAB.

**Figure 1:**
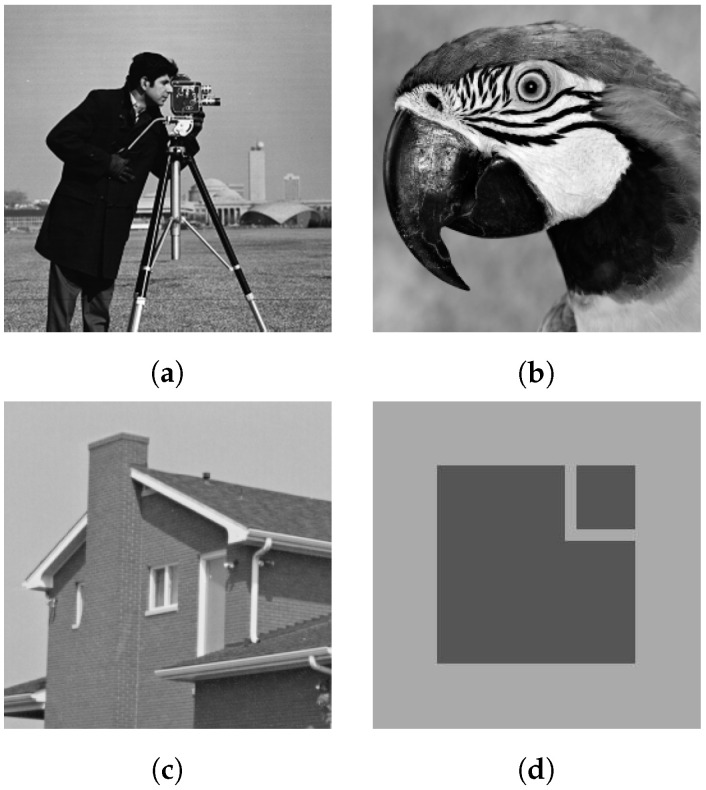
Original image; (**a**) “Cameraman”; (**b**) “Parrot”; (**c**) “House”; (**d**) “Square”.

We selected three 256 by 256 grayscale images, namely Parrot, Cameraman, Lenna, House, and Square, to test and compare our model with the AA model, the RLO model, and the ZWN model. The original images can be seen in Figure 1. We assess the quality of the recovered image by comparing it with the peak signal-to-noise ratio (PSNR) [32], defined as follows:

$$PSNR=10\log_{10}\frac{\max_{i}\left\{{\left[u\right]}_{i},{\left[\tilde{u}\right]}_{i}\right\}}{\frac{1}{m}{\sum {}_{i=1}^{m}\left({\left[u\right]}_{i}-{\left[\tilde{u}\right]}_{i}\right)}^{2}},$$

where *u* and $\tilde{u}$ are the original image and the recovered image, respectively. The PSNR measures the similarity between the recovered image and the original image in terms of pixel values. A higher PSNR indicates a higher quality of recovery. We employ the relative error as the stopping criterion, which is defined as follows:

$$\frac{{∥u^{k}-u^{k+1}∥}_{2}}{{∥u^{k}∥}_{2}}\leq 5\times 10^{4},$$

or the maximum number of iterations 500 reached.

### 4.1. Image Denoising

**Figure 2:**
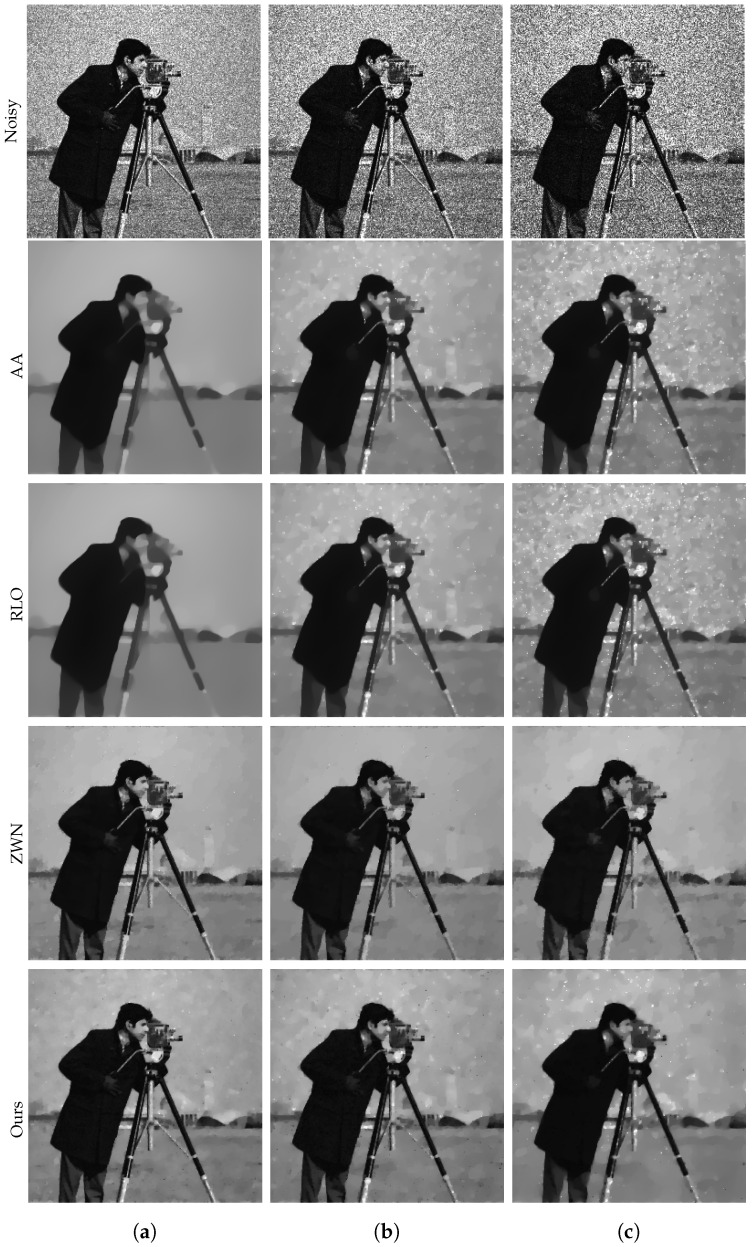
The results of different models removing multiplicative noise. Row 1: Noisy Cameraman, Row 2–5: the images recovered by the AA, RLO, ZWN, and our model, respectively. Column (**a**) to (**c**): noise level (**a**): L=20 (AA: λ=5, RLO: $\lambda =10^{-7}$, ZWN: $\alpha_{2}=0.001$, our: α=0.005), (**b**): L=10 (AA: λ=5, RLO: $\lambda =10^{-6}$, ZWN: $\alpha_{2}=0.002$, our: α=0.01), (**c**): L=6 (AA: λ=5, RLO: $\lambda =10^{-6}$, ZWN: $\alpha_{2}=0.002$, our: α=0.06).

**Figure 3:**
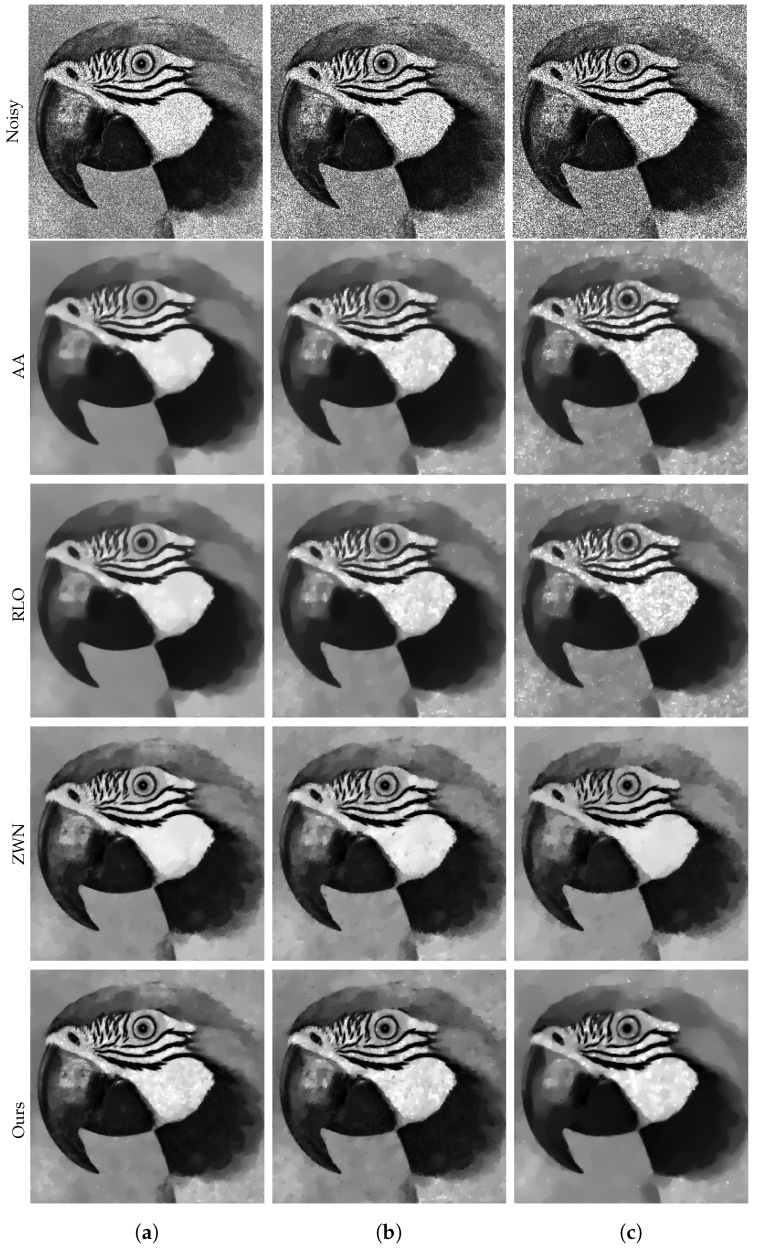
The results of different models removing multiplicative noise. Row 1: Noisy Parrot, Row 2–5: the images recovered by the AA, RLO, ZWN, and our model, respectively. Column (**a**) to (**c**): noise level (**a**): L=20 (AA: λ=0.7, RLO: $\lambda =5\times 10^{-6}$, ZWN: $\alpha_{2}=8\times 10^{-4}$, our: α=0.008), (**b**): L=10 (AA: λ=0.3, RLO: $\lambda =3\times 10^{-6}$, ZWN: $\alpha_{2}=0.001$, our: α=0.03), (**c**): L=6 (AA: λ=1, RLO: $\lambda =5\times 10^{-7}$, ZWN: $\alpha_{2}=0.002$, our: α=0.13).

**Figure 4:**
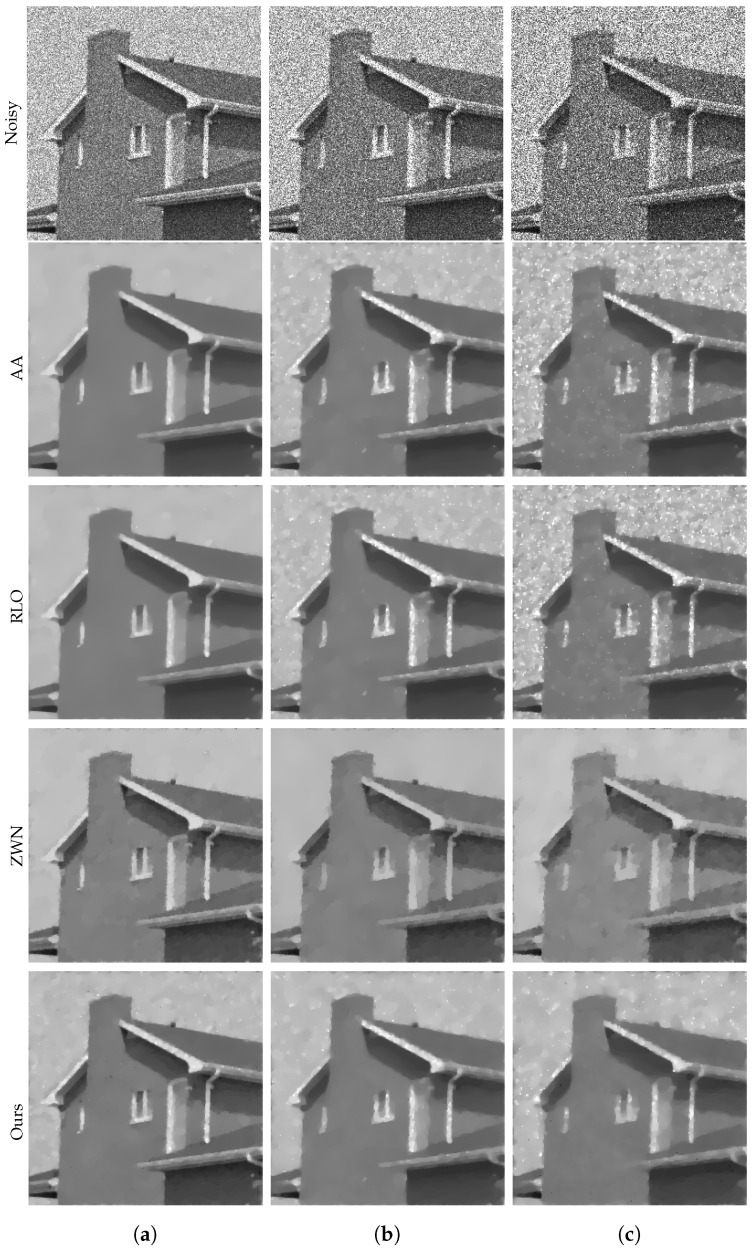
The results of different models removing multiplicative noise. Row 1: Noisy House, Row 2–5: the images recovered by the AA, RLO, ZWN, and our model, respectively. Column (**a**) to (**c**): noise level (**a**): L=20 (AA: λ=5, RLO: $\lambda =10^{-5}$, ZWN: $\alpha_{2}=9\times 10^{-4}$, our: α=0.007), (**b**): L=10 (AA: λ=5, RLO: $\lambda =4\times 10^{-6}$, ZWN: $\alpha_{2}=7\times 10^{-5}$, our: α=0.05), (**c**): L=6 (AA: $\lambda =8\times 10^{-4}$, RLO: λ=5, ZWN: $\alpha_{2}=5\times 10^{-5}$, our: α=0.07).

**Figure 5:**
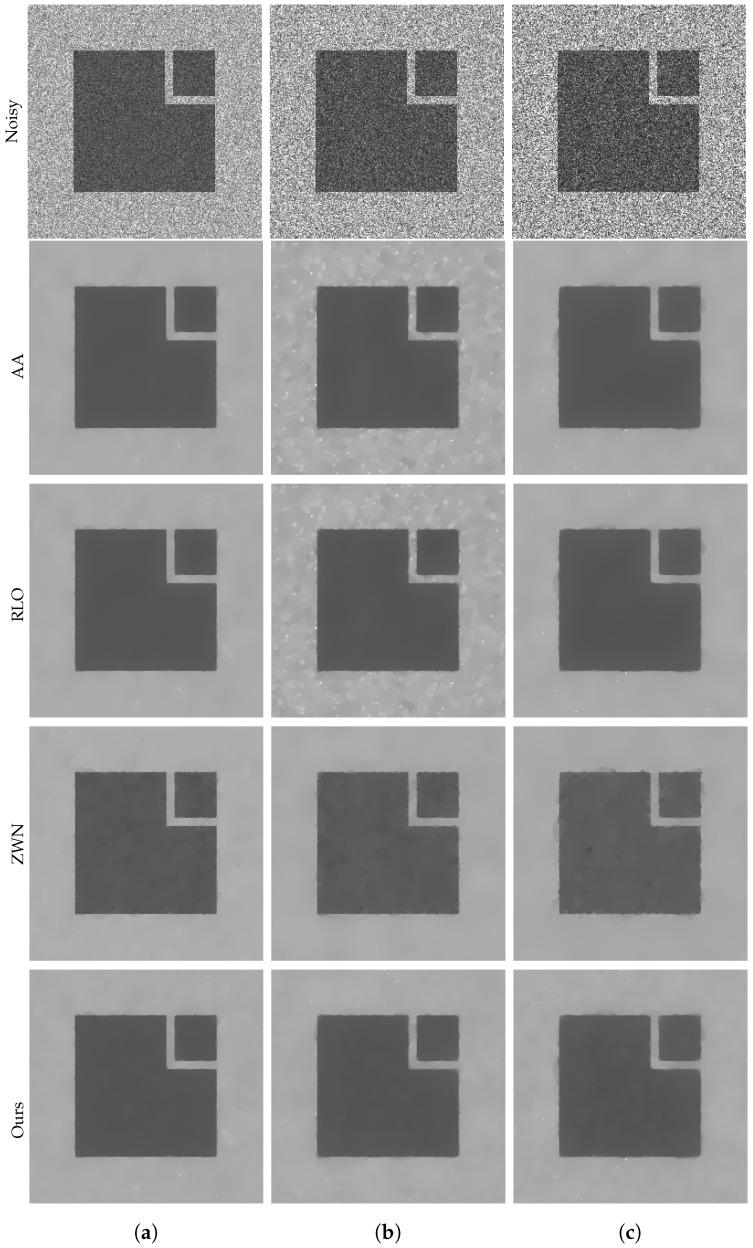
The results of different models removing multiplicative noise. Row 1: Noisy Square, Row 2–5: the images recovered by the AA, RLO, ZWN, and our model, respectively. Column (**a**) to (**c**): noise level (**a**): L=20 (AA: λ=5, RLO: $\lambda =10^{-3}$, ZWN: $\alpha_{2}=0.001$, our: α=0.03), (**b**): L=10 (AA: $\lambda =10^{-3}$, RLO: λ=5, ZWN: $\alpha_{2}=0.002$, our: α=0.09), (**c**): L=6 (AA: $\lambda =6\times 10^{-6}$, RLO: λ=5, ZWN: $\alpha_{2}=0.003$, our: α=0.3).

In this subsection, we focus on demonstrating the effectiveness of our proposed model for denoising purposes only. We consider the scenario that the original images are corrupted by multiplicative noise, following a Gamma distribution with L=20, L=10, and L=6, respectively. The denoising results are presented in Figure 2, Figure 3, Figure 4 and Figure 5. Moreover, we provide the corresponding PSNR values, computation time, and number of iterations in Table 1. The results consistently demonstrate that both the ZWN model and our model outperform the AA model and the RLO model across all metrics. Our model exhibits the highest PSNR values for most images at L=20, indicating its superior capability in reducing low levels of noise. Although at L=10 and L=6, our model still performs better than the AA model and the RLO model, it does not consistently surpass the ZWN model. This implies that our model is robust and effective in denoising images under various noise conditions, but it can be further enhanced to handle higher levels of noise. Therefore, we can confidently assert that our model offers significant advantages over existing models in image denoising.

### 4.2. Image Deblurring and Denoising

**Figure 6:**
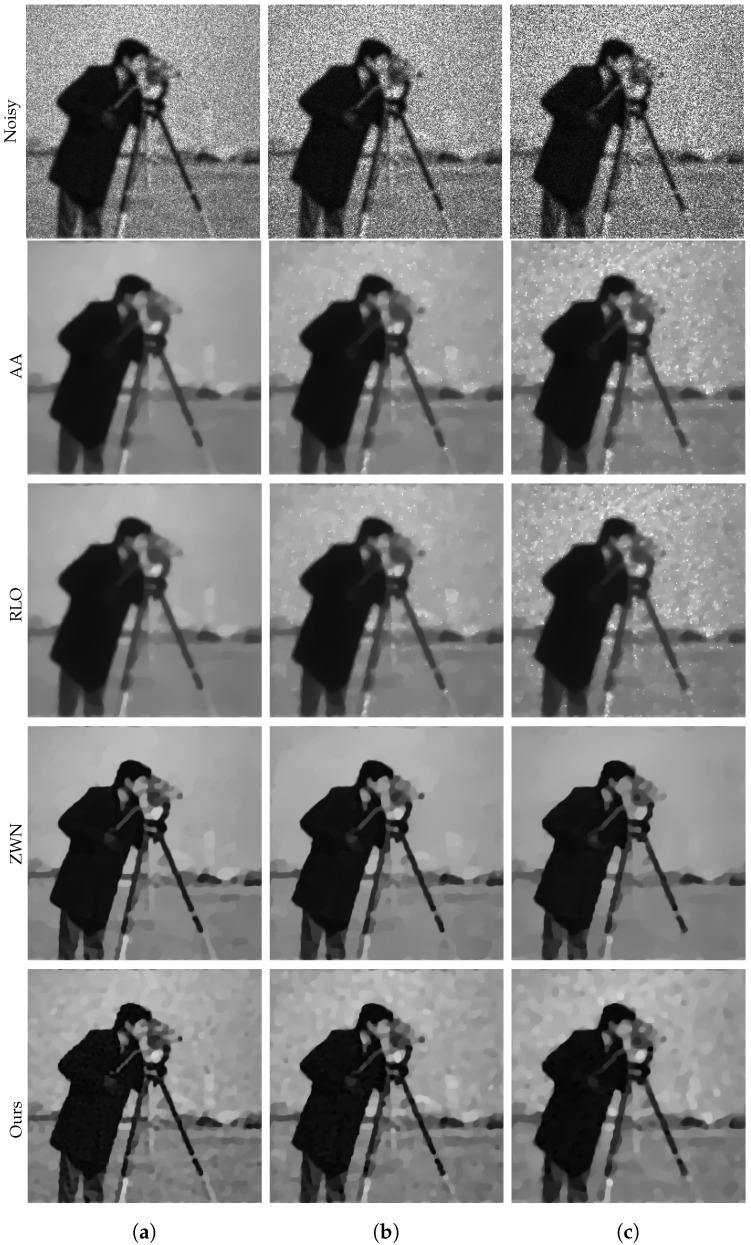
The results of different models removing multiplicative noise and blur. Row 1: Noisy Cameraman, Row 2–5: the images recovered by the AA, RLO, ZWN, and our model, respectively. Column (**a**) to (**c**): noise level (**a**): L=20 (AA: λ=5, RLO: $\lambda =3\times 10^{-7}$, ZWN: $\alpha_{2}=6\times 10^{-4}$, our: α=0.001), (**b**): L=10 (AA: λ=5, RLO: $\lambda =5\times 10^{-6}$, ZWN: $\alpha_{2}=0.001$, our: α=0.004), (**c**): L=6 (AA: λ=5, RLO: $\lambda =9\times 10^{-4}$, ZWN: $\alpha_{2}=0.002$, our: α=0.019).

**Figure 7:**
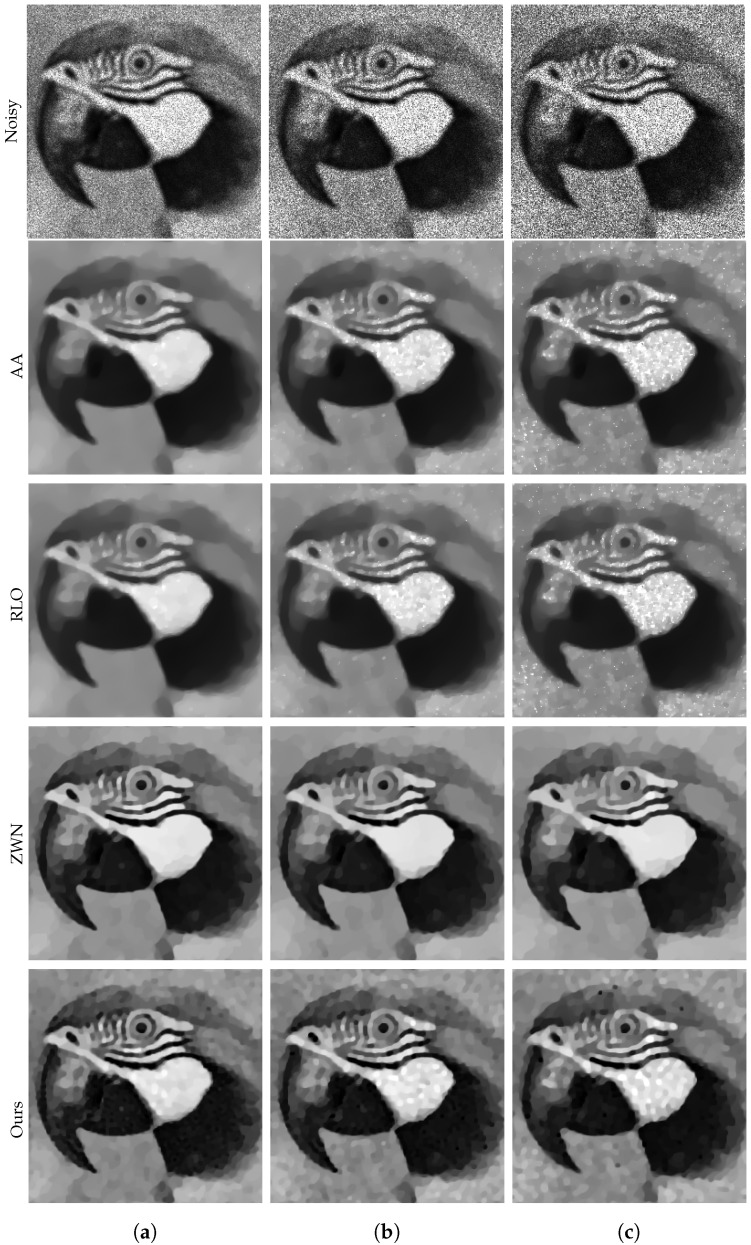
The results of different models removing multiplicative noise and blur. Row 1: Noisy Parrot, Row 2–5: the images recovered by the AA, RLO, ZWN, and our model, respectively. Column (**a**) to (**c**): noise level (**a**): L=20 (AA: λ=5, RLO: $\lambda =9\times 10^{-5}$, ZWN: $\alpha_{2}=10^{-4}$, our: $\alpha =6\times 10^{-4}$), (**b**): L=10 (AA: λ=5, RLO: $\lambda =7\times 10^{-7}$, ZWN: $\alpha_{2}=8\times 10^{-4}$, our: α=0.003), (**c**): L=6 (AA: λ=5, RLO: $\lambda =4\times 10^{-6}$, ZWN: $\alpha_{2}=0.001$, our: α=0.019).

**Figure 8:**
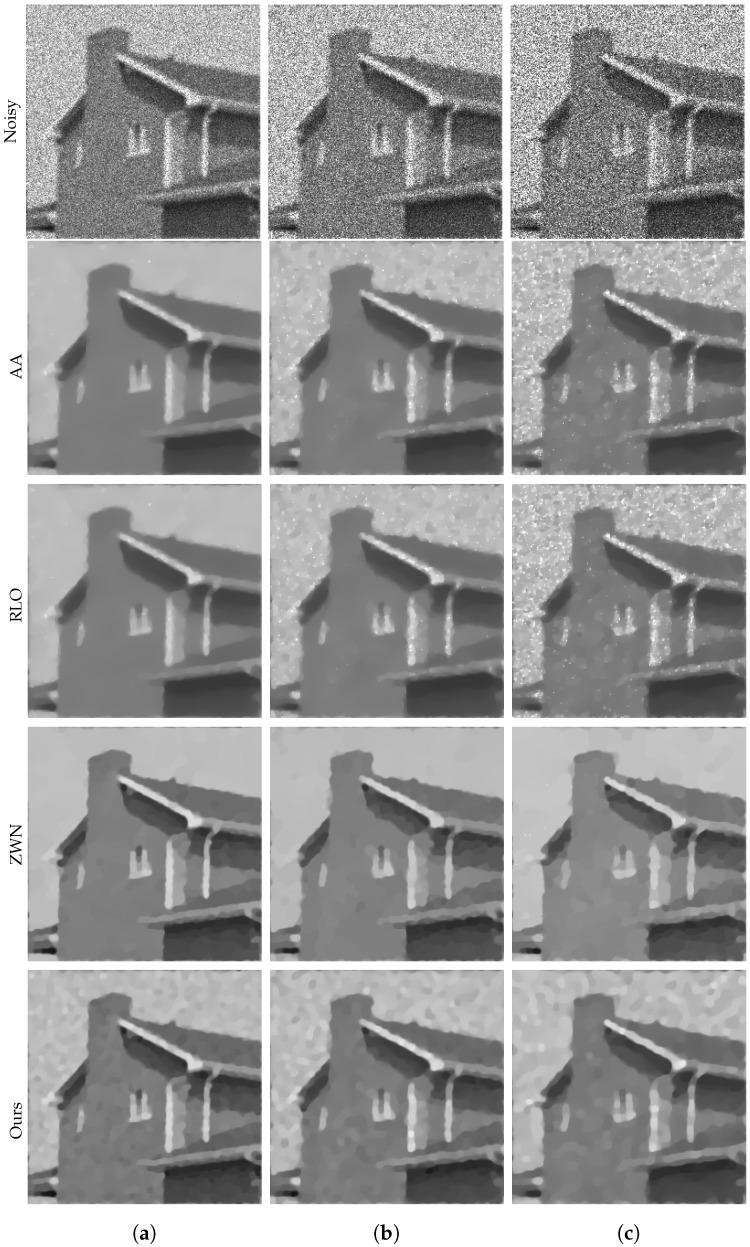
The results of different models removing multiplicative noise and blur. Row 1: Noisy House, Row 2–5: the images recovered by the AA, RLO, ZWN, and our model, respectively. Column (**a**) to (**c**): noise level (**a**): L=20 (AA: λ=5, RLO: $\lambda =2\times 10^{-4}$, ZWN: $\alpha_{2}=5\times 10^{-4}$, our: α=0.002), (**b**): L=10 (AA: λ=5, RLO: λ=0.01, ZWN: $\alpha_{2}=9\times 10^{-4}$, our: α=0.01), (**c**): L=6 (AA: λ=5, RLO: $\lambda =10^{-6}$, ZWN: $\alpha_{2}=0.001$, our: α=0.04).

**Figure 9:**
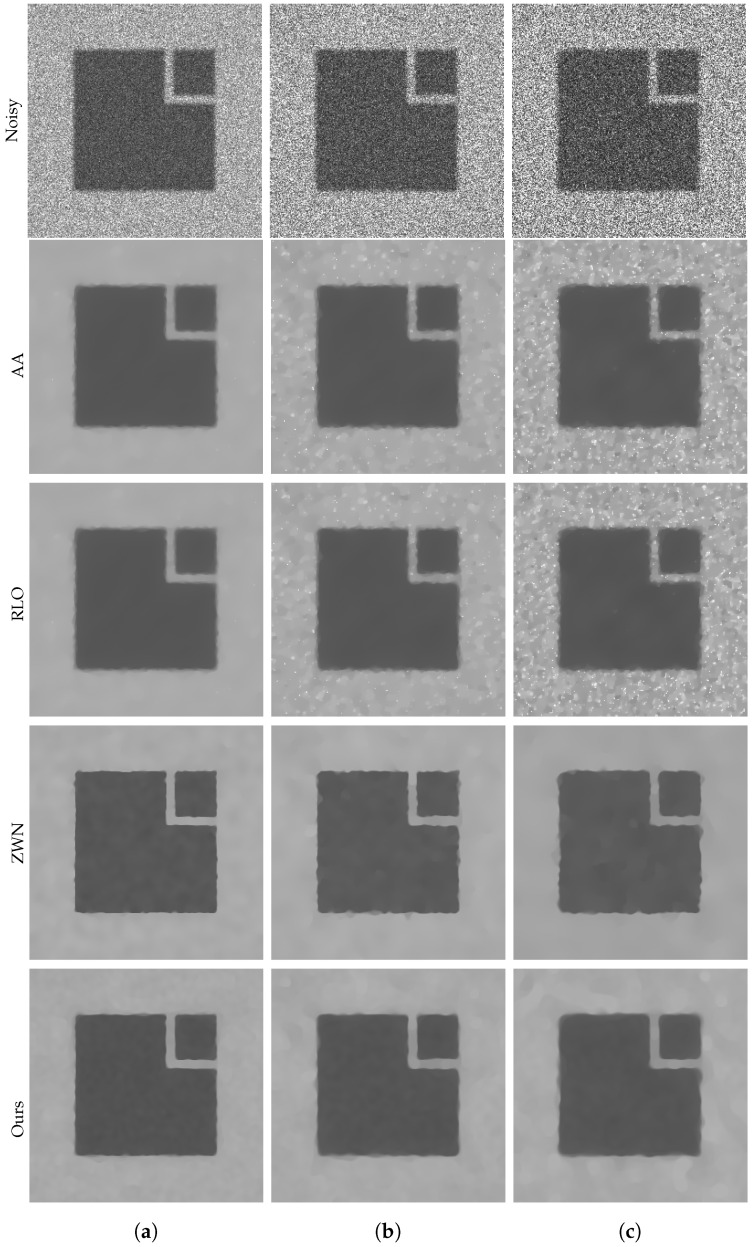
The results of different models removing multiplicative noise and blur. Row 1: Noisy Square, Row 2–5: the images recovered by the AA, RLO, ZWN, and our model, respectively. Column (**a**) to (**c**): noise level (**a**): L=20 (AA: λ=5, RLO: $\lambda =10^{-4}$, ZWN: $\alpha_{2}=7\times 10^{-4}$, our: α=0.006), (**b**): L=10 (AA: λ=5, RLO: λ=0.05, ZWN: $\alpha_{2}=0.001$, our: α=0.05), (**c**): L=6 (AA: $\lambda =6\times 10^{-5}$, RLO: λ=5, ZWN: $\alpha_{2}=0.002$, our: α=0.6).

In this subsection, we present the performance of our proposed model in the task of image deblurring and denoising. Our model is designed to effectively restore images that are corrupted by both multiplicative noise and blur. We evaluate our model on three distinct levels of noise, where the noise follows a Gamma distribution with L=20, L=10, and L=6, respectively. To simulate the blurring effect, we employ a 7×7 Gaussian kernel with zero mean and a standard deviation of 2. We compare our model with three other models in terms of deblurring and denoising, and present the experimental results in the form of data and images. The deblurring results are illustrated in Figure 6, Figure 7, Figure 8 and Figure 9. The corresponding PSNR values, execution times, and number of iterations are displayed in Table 2. The results indicate that our model consistently achieves the highest PSNR values in all cases, demonstrating its superior performance in denoising and deblurring. However, it is worth noting that our model also exhibits longer execution times in most cases, suggesting that it is computationally expensive. On the other hand, the ZWN model consistently demonstrates the lowest execution times in all cases, indicating its exceptional speed. Nonetheless, it also consistently yields the lowest PSNR values, suggesting subpar performance in denoising and deblurring. The AA model and RLO model exhibit similar PSNR values and execution times across all cases, indicating comparable performance and speed. Additionally, the results demonstrate that as the value of *L* decreases, the PSNR values also decrease and the running time increases for all models and images. This observation suggests that lower *L* values correspond to higher levels of noise and blur, making the denoising and deblurring tasks more challenging and time-consuming.

### 4.3. Real Image Denoising

**Figure 10:**
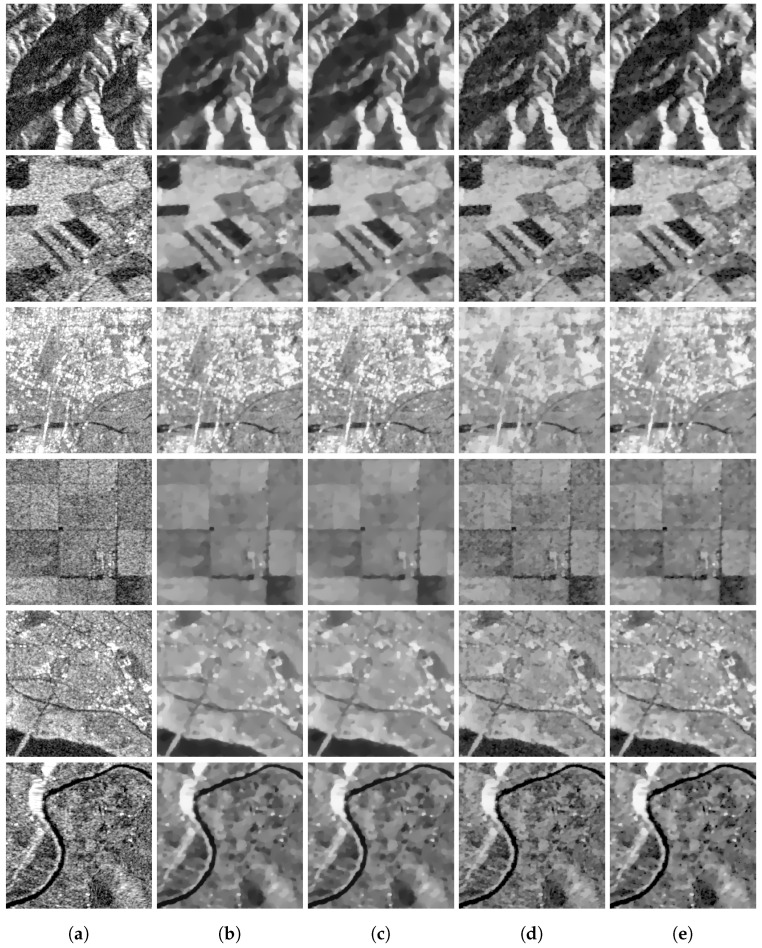
Comparison of different models for denoising real images. The first column (**a**) shows the original noisy images obtained from a dataset of SAR and optical image patches. The second column (**b**) shows the images recovered by the AA model (λ=0.05). The third column (**c**) shows the images recovered by the RLO model (λ=0.0009). The fourth column (**d**) shows the images recovered by the ZWN model ($\alpha_{2}=0.001$). The fifth column (**e**) shows the images recovered by our proposed model (α=0.05).

In this subsection, we evaluate the performance of our approach on real images that are affected by multiplicative noise, also known as speckle noise. This type of noise occurs in coherent imaging systems such as SAR and ultrasound imaging. We use the same regularization parameters to remove different images in order to observe the robustness of different models, since the selection of the regularization parameters is the most difficult point in removing real images. We selected five images with different features from a dataset that consists of 282,384 pairs of corresponding SAR and optical image patches acquired by the Sentinel-1 and Sentinel-2 satellites. The dataset was downloaded from the following URL: https://mediatum.ub.tum.de/1436631. Figure 10 shows the tested images and the images denoised by using four different methods. Among the four models we compared, the AA model and the RLO model produced similar results, but they tended to over-smooth the images and lose a lot of edge information. On the other hand, our model and the ZWN model preserved the edges better and achieved more visually pleasing results. These two models also showed a similar performance in removing noise from different images, even though we used the same regularization parameters for all of them.

## 5. Conclusions

In this paper, we proposed a new model based on the ZWN model that improves the sparsity of the solution and reduces the number of regularization parameters by incorporating an equality constraint. To solve the proposed model, we applied the popular ADMM method. The effectiveness and efficiency of our model were evaluated on various public datasets and compared with the ZWN model, along with the RLO and the AA methods. The experimental results demonstrated that our model achieves superior or comparable performance in terms of PSNR and subjective visual assessment. Moreover, we also tested our model on real images that are affected by multiplicative noise. We showed that our model can handle this type of noise well and produce more visually pleasing results than the other methods.

Though the total variation technique effectively preserves edges and removes noise, it often creates undesirable staircase artifacts, particularly in areas with flat regions or smooth gradients in an image. To mitigate these artifacts, numerous improvements and modifications have been made to the traditional total variation model. These include approaches such as higher-order total variation, total generalized variation (TGV), and infimal convolution of total variation (ICTV), among others. In our future work, we aim to study these modifications to enhance the performance of total variation regularization and reduce the occurrence of staircase artifacts, thereby improving the overall quality of restored or reconstructed images. This could potentially lead to improved performance in various applications such as medical imaging, remote sensing, and computer vision.

## Figures and Tables

**Table 1:** Comparison of PSNR values (dB), number of iterations, and time (s) of different methods in the case of denoising.

Image	Model	L=20			L=10			L=6		
		PSNR	Time	Iter	PSNR	Time	Iter	PSNR	Time	Iter
Cameraman	AA	24.55	0.83	500	24.15	**0.78**	500	22.83	**0.80**	500
	RLO	24.40	**0.82**	500	24.02	0.83	500	22.75	0.81	500
	ZWN	26.43	1.42	**162**	24.58	1.30	**148**	**23.61**	1.08	**119**
	Ours	**26.50**	1.49	251	**24.60**	1.80	304	23.44	2.17	366
Parrot	AA	24.46	**0.80**	500	23.79	**0.81**	500	22.31	**0.84**	500
	RLO	24.40	0.81	500	23.78	**0.81**	500	22.28	0.92	500
	ZWN	26.80	1.26	**145**	**24.90**	1.34	**149**	**23.53**	1.12	**126**
	Ours	**26.84**	1.33	219	24.89	1.82	309	23.37	2.26	381
House	AA	27.32	0.93	500	26.15	0.83	500	23.04	0.80	500
	RLO	27.28	0.84	500	26.13	0.83	500	23.05	0.82	500
	ZWN	27.91	**0.66**	**64**	25.44	**0.28**	**27**	23.74	**0.31**	**29**
	Ours	**28.24**	1.21	189	**26.40**	1.50	255	**24.82**	2.03	345
Square	AA	**34.36**	0.97	500	29.32	0.83	500	23.18	0.87	500
	RLO	34.33	0.87	500	29.35	0.82	500	23.22	0.85	500
	ZWN	33.02	**0.29**	**26**	30.57	**0.29**	**42**	27.84	**0.39**	**26**
	Ours	34.21	0.95	136	**31.61**	1.06	180	**29.94**	1.34	136

**Table 2:** Comparison of PSNR values (dB), number of iterations, and time (s) of different methods in the case of deblurring.

Image	Model	L=20			L=10			L=6		
		PSNR	Time	Iter	PSNR	Time	Iter	PSNR	Time	Iter
Cameraman	AA	21.33	0.99	500	21.23	1.10	500	20.62	1.03	500
	RLO	21.27	0.97	500	21.17	1.01	500	20.58	0.92	500
	ZWN	22.93	**0.36**	**38**	**22.43**	**0.37**	**39**	21.75	**0.44**	**42**
	Ours	**22.99**	0.50	69	22.37	0.79	106	**21.80**	1.28	131
Parrot	AA	20.41	1.00	500	20.30	1.13	500	19.71	1.03	500
	RLO	20.35	0.93	500	20.25	0.97	500	19.67	0.98	500
	ZWN	22.28	**0.43**	**44**	21.45	**0.42**	**46**	20.64	**0.48**	**49**
	Ours	**22.35**	0.72	77	**21.65**	0.79	106	**20.97**	0.89	131
House	AA	24.19	0.97	500	23.73	1.08	500	22.06	1.01	500
	RLO	24.17	0.96	500	23.71	1.01	500	22.06	1.00	500
	ZWN	**25.85**	**0.46**	**50**	24.47	**0.69**	**73**	22.65	**0.79**	**88**
	Ours	25.67	0.61	76	**24.67**	0.70	98	**24.00**	**0.79**	120
Square	AA	28.31	1.00	500	26.49	1.01	500	22.51	1.17	500
	RLO	28.30	0.96	500	26.48	0.95	500	22.52	1.14	500
	ZWN	30.07	**0.35**	**37**	28.47	**0.29**	**28**	25.95	**0.32**	**31**
	Ours	**31.55**	0.81	118	**28.61**	0.78	113	**27.76**	0.87	117

## Data Availability

The code used to generate the results and figures is available in a Github repository at https://github.com/hhaaoo1331/Simplified_multicative_noise (accessed on 24 August 2023).
